# Supplementary material for: Tumor evolutionary directed graphs and the history of chronic lymphocytic leukemia
Source: eLife. 2014 Dec 11;3:e02869. doi: 10.7554/eLife.02869 (PMC4308685; doi:10.7554/eLife.02869)
Supplement: Supplementary file 1. — PCR primers and conditions. DOI: http://dx.doi.org/10.7554/eLife.02869.020 [file elife02869s001.docx]

**PCR and sequencing conditions**

PCR reactions were performed with the Go Taq Hot Start polymerase (Promega, Madison, WI, USA), on 50 ng of genomic DNA under the following conditions: denaturing step at 95°C for 3 min followed by 38 cycles at 95°C (30 seconds per cycle), annealing step at 58°C (40 seconds per cycle), and extension at 72°C (40 seconds per cycle), in a Verity 96 well thermal cycler (Applied Biosystems). The final concentrations of each PCR primer were 0.17 μM in a final volume of 30 microliters. Products were resolved on 2% agarose gels by electrophoresis and visualized after staining with ethidium bromide. Purified PCR products were directly sequenced with the ABI PRISM BigDye Terminator v1.1 Cycle Sequencing Kit (Applied Biosystems) using the ABI PRISM 3100 Genetic Analyzer (Applied Biosystems). The final concentrations of each sequencing primer were 0.16 μM in a final volume of 20 microliters, and all sequencing reactions were performed using the same primers of PCR. Specific sequences of PCR and sequencing primers are reported in table S1.

**Table S1. PCR and sequencing primers**

| **Gene** | **Exon** | **Forward Primer** | **Reverse Primer** |
| --- | --- | --- | --- |
| *TP53* | 4 | CGTTCTGGTAAGGACAAGGG | AAGGGTGAAGAGGAATCCCA |
| *TP53* | 5 | GTTTGTTTCTTTGCTGCCGT | AGAGGCCTGGGGACCCT |
| *TP53* | 6 | GACAGGGCTGGTTGCCC | TCATGGGGTTATAGGGAGGTC |
| *TP53* | 7 | CCTGCTTGCCACAGGTCT | GTGATGAGAGGTGGATGGGT |
| *TP53* | 8-9 | TGGGACAGGTAGGACCTGATT | GAGGCAAGGAAAGGTGATAAAA |
| *NOTCH1* | 34.1 | CCTCTGGTGATGGAACCTTG | AGGTAGCCATGGGGTGACTC |
| *NOTCH1* | 34.2 | GGCAAGAAGGTCCGCAAG | CGCAGAGGGTTGTATTGGTT |
| *NOTCH1* | 34.3 | TCCACCAGTTTGAATGGTCA | TCTCCTGGGGCAGAATAGTG |
| *NOTCH1* | 34.4 | CCACCACCACCACCACAG | AAGGCTTGGGAAAGGAAGC |
| *SF3B1* | 14 | TGGAAAGAAATGGTTGAAGA | AAGACCCTGTCTCCTAAAGAAAAA |
| *SF3B1* | 15 | TGCAGTTTGGCTGAATAGTTG | CCAATAGCCTTCAAGAAAGCAG |
| *SF3B1* | 16 | CAGAGGAAAGGTAAATCCACCA | AACCATGAAACATATCCAGTTTACA |
| *SF3B1* | 18 | TCCTTGGAAAAGCAGTCTAAAAG | GGTAACCCCCTGAGCATTTT |
| *MYD88* | 3 | CTAGGCAGGGGACTCTTGG | GCACAGCTAGGAGGAGATGC |
| *MYD88* | 5 | GTTGAAGACTGGGCTTGTCC | AGGAGGCAGGGCAGAAGTA |
| *BIRC3* | 6 | AATGCCTATACATTTTGTTGGTTT | AGACTGATATCAAATCCTTATGAAAAT |
| *BIRC3* | 7 | TGGAAGGAAGTTTGTGAGCA | AAGAGCGTATTTTCAATTGACTTAGA |
| *BIRC3* | 8 | GACTTCTGTTGCCTTGAAATGA | AGGCAGTTTGCTTCTTCAGTG |
| *BIRC3* | 9 | TCCCTTAGTGCAACAGGACA | AAGGAAACCAAATTAGGATAAAAGTT |
